# Continental Notes from a Holiday Correspondent

**Published:** 1894-09-08

**Authors:** 


### CONTINENTAL NOTES FROM A HOLIDAY CORRESPONDENT.

BONN.

The town of Bonn differs in many respects from its nearer neighbours on the Rhine. It is exceedingly old, having been the first Roman station on the Rhine, and the headquarters of several legions, but it has lately ceased to live merely on the memories of its ancient glory, and has taken a new lease of life and prosperity, and is rapidly increasing in size. Much of this increase is no doubt due to its rising commercial prosperity, but part is also due to the prosperity of its University. This was founded so lately as 1818, so is a mere infant among German universities as regards age, though not as regards size, for it is already attended by some thirteen or fourteen hundred students, and is generally looked on as in the front rank of seats of learning. The main University building was originally a palace, built a hundred years before the University was founded, and is a curious, rather dilapidated, old place. It is on the south side of the town, fronting a fine large grass plot, surrounded with old trees, reminding one of the "backs" at Cambridge. In this building are the class-rooms (except most of the medical ones), the library, containing nearly a quarter of a million volumes, the physical institute, museums, and ophthalmic institute. In the last Professor Siemisch and his assistants are to be found each morning at half-past eight during the summer months. Both the Professor and his chief assistant speak English. The patients are very numerous, as they come not only from Cologne, twenty miles off, but from all the populous country round Bonn and further up the Rhine. There are about 4,500 cases a year in the extern department. No operative work was going on during my visit, but many interesting cases were to be seen. Amongst these was a boy of ten with filtration of the vitreous, following cerebro-spinal meningitis; the eye was soft and shrinking.

On Mondays, Tuesdays, and Fridays, from eight to nine o'clock, Professor Siemisch lectures to a class of forty or fifty, and then examines cases. The Professor is most courteous to strangers, and seems anxious to show everything of interest to them, including, naturally, his well-known treatment of corneal ulcers by incision. The ophthalmic institute is, I believe, the only part of the medical school located in the main University building. The principal sections of the school are in a group of buildings at the north side of the town. These buildings are finely situated on an elevated tract of land, with plenty of open ground round them, and all enclosed in one boundary wall. They comprise a general hospital, a medical clinic, a surgical clinic, an obstetric and gynæcological clinic, and a pathological institute. All are equipped in the most perfect fashion, and it would be hard to find a more complete set of buildings in a provincial town. They cost about £150,000.

The women's clinic is under the care of Professor Kehrer and two resident assistants. The Professor talks English fairly well, and is pleased to see visitors. His antisepsis is the ordinary strict form seen in German hospitals. His name is best known to the profession in connection with his operation for amputation of the cervix.

The old Schloss on the north side of the town is devoted to the very fine natural history collections. A well-stocked botanical garden adjoins the Schloss, and across the road close by is the famous chemical laboratory, one of the best, if not the best, in the world. In it Hofmann's chief work was done. A fine anatomy building, built in 1872, stands beside the laboratory, and not far off are the buildings of the agricultural school and the physiological institute. To the visitor interested in medical education all these are worth seeing, but they form a sight calculated to arouse feelings of envy as well as admiration in those familiar with the poverty-struck condition of most of our provincial and even metropolitan schools. Here, as in other German cities, money seems to be lavished in the most profuse way on all seats of learning, whether scientific or literary, and one must acknowledge freely that the outlay is well repaid in the thoroughness of the education attained by Germans as by no other nation.

In concluding these "Continental Notes" allow me to recommend strongly any medical man in doubt about a tour, to follow some such course as has been indicated in these notes. For a young man specially, who wishes to see some work as well as amuse himself, no tour can be better than one embracing the places I have referred to. The L. C. and D. Railway gives a circular ticket from London, via Calais, Paris, Baden, Heidelberg, Frankfort, Wiesbaden, Ems, Cologne, Ostend and Dover, back to London, for £8 6s. first class, and £6 7s. second. Including the cost of this ticket a month's tour can be done comfortably for £30, and with economy for a good deal less. After it the medical tourist will return to work benefited not only in body, but having also benefitted in his professional knowledge, and that by personal observation in other lands, a much pleasanter and easier process than by reading foreign text books alone.

